# Synaptic polarity of the interneuron circuit controlling *C. elegans* locomotion

**DOI:** 10.3389/fncom.2013.00128

**Published:** 2013-10-02

**Authors:** Franciszek Rakowski, Jagan Srinivasan, Paul W. Sternberg, Jan Karbowski

**Affiliations:** ^1^Interdisciplinary Center for Mathematical and Computational Modeling, University of WarsawWarsaw, Poland; ^2^Worcester Polytechnic InstituteWorcester, MA, USA; ^3^Division of Biology, California Institute of TechnologyPasadena, CA, USA; ^4^Institute of Applied Mathematics and Mechanics, University of WarsawWarsaw, Poland; ^5^Institute of Biocybernetics and Biomedical Engineering, Polish Academy of SciencesWarsaw, Poland

**Keywords:** *C. elegans*, locomotory interneurons, synaptic polarity, locomotion, neural circuit modeling, optimization, laser ablations

## Abstract

*Caenorhabditis elegans* is the only animal for which a detailed neural connectivity diagram has been constructed. However, synaptic polarities in this diagram, and thus, circuit functions are largely unknown. Here, we deciphered the likely polarities of seven pre-motor neurons implicated in the control of worm's locomotion, using a combination of experimental and computational tools. We performed single and multiple laser ablations in the locomotor interneuron circuit and recorded times the worms spent in forward and backward locomotion. We constructed a theoretical model of the locomotor circuit and searched its all possible synaptic polarity combinations and sensory input patterns in order to find the best match to the timing data. The optimal solution is when either all or most of the interneurons are inhibitory and forward interneurons receive the strongest input, which suggests that inhibition governs the dynamics of the locomotor interneuron circuit. From the five pre-motor interneurons, only AVB and AVD are equally likely to be excitatory, i.e., they have probably similar number of inhibitory and excitatory connections to distant targets. The method used here has a general character and thus can be also applied to other neural systems consisting of small functional networks.

## Introduction

*Caenorhabditis elegans* nematode worms possess a very small nervous system composed of only 302 neurons connected by about 5000 chemical synapses and 3000 gap junctions (White et al., 1986). Because of its smallness a precise map of neural connections was possible to construct (White et al., 1986; Chen et al., 2006). This places *C. elegans* in a unique position among all other animals (Varshney et al., 2011), for which we have at best only rudimentary connectivity data to test various concepts regarding neural wiring and function (Cherniak, 1994; Karbowski, 2001, 2003; Chklovskii, 2004; Chen et al., 2006; Kaiser and Hilgetag, 2006). However, despite this achievement we still have a very limited knowledge about the nature of most of the worm's synaptic connections, i.e., whether they are excitatory or inhibitory.

The simplicity of the *C. elegans* nervous system does not preclude these worms from executing various non-trivial behaviors such as locomotion, feeding, mating, chemotaxis, etc. (Hobert, 2003; de Bono and Maricq, 2005). To understand the neural basis of these behaviors requires some information not only about the pattern and strength of the connections but also about the type of their synapses. The same neural circuit can perform different functions depending on the signs of synaptic polarities it contains. Specifically, circuits in which excitatory synapses dominate can sometimes become epileptic. On the other hand, networks with only inhibitory connections could be silent, and therefore in many situations useless. Thus, it may seem that some sort of an intermediate regime is necessary for a proper functioning of the nervous system (van Vreeswijk and Sompolinsky, 1996). For example, it was proposed that mammalian cortical networks operate in a dynamic state in which excitation is effectively balanced by inhibition (Haider et al., 2006; Vogels et al., 2011), although anatomical number of excitatory connections dominates over inhibitory in the cerebral cortex (DeFelipe et al., 2002). For nematode worms, a similar issue has been addressed only sporadically. On a modeling level, in the context of a tap withdrawal circuit (Wicks et al., 1996), and experimentally, studying genes that influence the ratio of excitatory to inhibitory signaling (Jospin et al., 2009). We think that this topic deserves more attention both theoretical and experimental, if we are to understand the functioning of worm's circuits (Gray et al., 2005; Sengupta and Samuel, 2009; Ha et al., 2010).

Movement direction in *C. elegans* is governed by five distinct locomotory command interneurons (AVB, PVC, AVA, AVD, AVE), each in two copies (left and right). All of these 10 interneurons directly connect a downstream group of dorsal and ventral body wall excitatory motor neurons (Chalfie et al., 1985). The topology of connections between the command interneurons is well known (White et al., 1986; Chen et al., 2006), however, their synaptic polarities are not. Conventional thinking is that AVB and PVC control forward motion, while AVA, AVD, AVE control backward motion (Chalfie et al., 1985). This reasoning is based on the fact that the former interneurons connect mainly motor neurons of type B [experimentally shown to be critical for forward locomotion (Haspel et al., 2010)], whereas the latter interneurons connect mostly type A motor neurons [ruling the backward motion (Haspel et al., 2010)]. However, this simple locomotory picture, relying on a single neuron function doctrine may turn out to be too simplistic. Indeed, many laser ablation experiments show that removal of both AVB and PVC reduces forward motion, but does not abolish it completely (see below). Similarly, worms lacking the “backward” interneurons AVA, AVD, and AVE exhibit a comparable frequency of reversals as intact wild type (WT) worms (Piggott et al., 2011). Moreover, the major backward interneuron AVA makes also connections (both synaptic and gap junctions) with the forward B motor neurons (Chen et al., 2006). Thus, perhaps the decision to move in a particular direction is generated by a collective activity of all command interneurons, rather than by an activity of a particular interneuron or a particular connection.

Our aim is to investigate the problem of decision making for movement direction in *C. elegans* on the level of its interneuron network. The main question we pose is how two antagonistic behaviors, i.e., forward and backward motions, can be controlled by the same circuit of mutually coupled pre-motor interneurons. A strictly related to this question is the problem of synaptic polarities of these interneurons and the input pattern they receive. Specifically, by applying structural perturbations to the circuit we want to determine, using mathematical modeling, which combination of synaptic polarities (together with an input pattern) gives the best match to the experimentally observed locomotory output of *C. elegans*. This knowledge allows us to answer a question about a relative influence of inhibition and excitation in the command interneuron circuit. Moreover, this approach provides an insight about a degree of interneuron collectiveness in choosing the direction of motion.

## Results

### The command interneuron circuit for *C. elegans* locomotion

**Figure 1:**
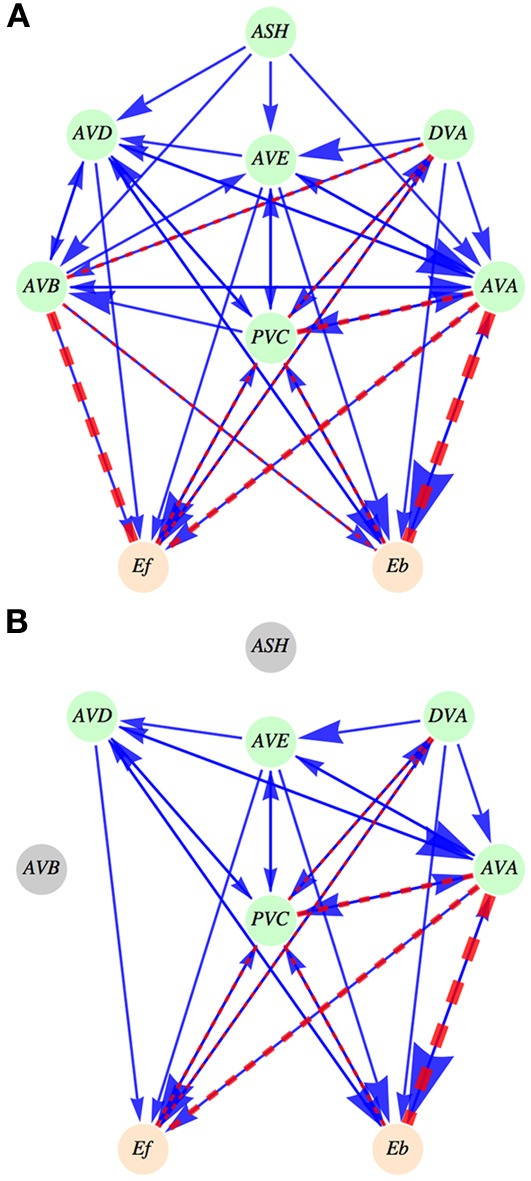
**Schematic diagram of the interneuron locomotory circuit. (A)** Intact circuit. ASH neuron is an upstream neuron that provides synaptic input to the locomotory interneurons. The output coming from the six neurons (five interneurons and DVA) feeds the activities of motor neurons, represented by *E*_*f*_ (controlling forward motion) and by *E*_*b*_ (controlling backward motion). Synaptic connections are shown as solid arrows (blue), and gap junctions are represented by dashed lines (red). The magnitude of an arrow and the width of a dashed line are indicators of the strength of synaptic and gap junction connections, respectively. **(B)** An example of an ablated circuit, in which ASH and AVB neurons are removed. Note that this leads to the removal of all connections (synaptic and electric) coming out from these neurons. Such ablations not only change the circuit architecture but also modify its activity output.

To simplify data analysis and mathematical modeling we grouped the left and right members of each locomotor circuit neuron as one model neuron. Thus, in our circuit controlling worm's motion there are five command interneurons, one distinguished upstream polymodal sensory interneuron called ASH, and a modulatory neuron DVA (Figure 1A). A recent study (Li et al., 2006) indicates that DVA plays a role of a sensory neuron in locomotion. We included this neuron explicitly in the circuit, since it has direct connections with body wall motor neurons (Chen et al., 2006), similar to five command interneurons. Because of this similarity, we want to investigate whether DVA can serve additionally as a command interneuron.

The neurons in the circuit are modeled as a single passive compartment with leak conductance. Connections between neurons are either by chemical synapses (of unknown polarity) or by electric synapses known as gap junctions. Chemical synapses transmit signals using graded transmission. The strength of the connection between two arbitrary neurons is proportional to the number of anatomical contacts between them determined from the empirical data (Chen et al., 2006). Additionally, each pre-motor interneuron receives a constant in time excitatory input from upstream (mostly head) neurons, which can be either weak or strong (this is variable in the model). Overall, our model captures long-term averages in neural activities that are associated with average locomotory output in *C. elegans*. All the assumptions made in the model and equations describing activities of all neurons are presented in Materials and Methods and in Supplementary Information. The main parameters used in the model are described in Table 1.

**Table 1:** **The main parameters used in the model**.

**Symbol**	**Value or range**	**Description**
*q*_*s*_	0.03–0.6 nS (optimized)	Maximal conductance of a single synapse
*q*_*e*_	0.03–0.5 nS (optimized)	Conductance of a single gap junction
θ	45 mV (fixed)	Renormalized threshold for synaptic activation
γ	0.15 mV^−1^ (fixed)	Steepness of synaptic activation
σ	4–12 mV (variable)	Amplitude of strong input
*z*_*i*_	0 or 1	Binary variable indicating the presence of strong input
κ	0.2–0.75 (variable)	Excitation level of ASH neuron
η	0.1–2.0 mV (optimized)	Noise in the system

In an intact circuit for WT worms fluctuations in interneuron activities control forward and backward motion, and the distribution of these activities determines the relative proportion of forward and backward motion (roughly three to one). We wanted to perturb the system and investigate its corresponding output by performing laser ablations of selected neurons in the locomotory circuit. We reasoned that a gradual removal of neurons from this circuit (Figure 1B; see Materials and Methods) would not only affect its physical structure, but also would redistribute the remaining neurons activities, which in turn, should modify the worm's locomotory behavior. In particular, the pattern of interneuron activities in the circuit should change, altering the ratio of times spent in forward and backward motion. Thus, associating experimental average times of forward and backward locomotion for every ablation type with changes in the average activity levels of the circuit model for fixed sensory inputs can allow us to predict synaptic polarities of the interneurons.

### Experimental results

We performed single, double, triple, and quadruple ablations in the interneuron circuit. In total, we generated 17 types of ablation and recorded corresponding mean times the worms spent in forward (*T*_*f*_) and backward (*T*_*b*_) motion, as well as in stopped phase (*T*_*s*_). These experimental results are presented in Table 2. From all the ablations executed, only removal of ASH and PVC neurons increase the time spent in forward locomotion in relation to WT. This is an indication that these particular interneurons have a definitely negative influence on the forward direction, and for that reason are likely to be inhibitory (see below). All other removals have a detrimental effect on the duration of forward motion, even those traditionally associated with backward motion (AVA, AVD). In particular, the ablation of AVA has the most dramatic effect on *T*_*f*_, leading to its 10-fold reduction in comparison to WT, although the reversal frequency increases only mildly by a factor of 2. Moreover, the AVA ablated worms, including their multiple ablations, spent a lot of time not moving (stopped phase), far more than WT and worms with other types of ablation. For instance, for the combined ablation ASH + AVA + AVB, we find the largest stopped mean time of 1.16 s.

**Table 2:** **Experimental data of the impact of neuron ablation on *C. elegans* locomotion**.

**Ablation type**	**Forward time *T*_*f*_ (s)**	**Backward time *T*_*b*_ (s)**	**Stopped time (s)**	**Reversals (miyn^−1^)**
Mock ablated (WT, *N* = 43)	8.98 ± 0.57	2.80 ± 0.27	0.26 ± 0.01	5.29 ± 0.27
ASH (*N* = 14)	12.6 ± 1.67	0.93 ± 0.17	0.27 ± 0.01	3.79 ± 0.80
AVA (*N* = 11)	0.71 ± 0.09	0.53 ± 0.04	0.60 ± 0.05	10.3 ± 0.56
AVB (*N* = 8)	2.26 ± 0.40	2.14 ± 0.23	0.38 ± 0.02	6.10 ± 0.64
AVD (*N* = 4)	4.23 ± 1.80	3.12 ± 0.36	0.31 ± 0.04	3.50 ± 0.31
DVA (*N* = 22)	1.51 ± 0.18	1.23 ± 0.08	0.44 ± 0.02	10.0 ± 0.57
PVC (*N* = 12)	12.0 ± 1.81	1.89 ± 0.39	0.29 ± 0.02	5.46 ± 0.74
ASH + AVA (*N* = 7)	1.91 ± 0.42	0.85 ± 0.20	0.52 ± 0.06	5.18 ± 0.90
ASH + AVB (*N* = 12)	2.05 ± 0.47	2.04 ± 0.43	0.42 ± 0.06	6.92 ± 1.08
AVA + AVB (*N* = 9)	0.56 ± 0.14	0.46 ± 0.06	0.89 ± 0.17	10.1 ± 1.36
AVA + PVC (*N* = 11)	4.09 ± 0.91	0.67 ± 0.14	0.37 ± 0.04	9.78 ± 0.71
AVB + PVC (*N* = 5)	0.91 ± 0.24	1.19 ± 0.19	0.44 ± 0.08	15.0 ± 3.52
DVA + PVC (*N* = 19)	2.18 ± 0.21	1.35 ± 0.08	0.40 ± 0.02	11.6 ± 0.57
ASH + AVA + AVB (*N* = 8)	0.75 ± 0.24	0.52 ± 0.11	1.16 ± 0.26	6.47 ± 0.83
AVA + AVB + PVC (*N* = 8)	0.93 ± 0.33	0.47 ± 0.12	0.87 ± 0.21	6.10 ± 0.87
AVB + AVD + PVC (*N* = 5)	1.33 ± 0.31	0.94 ± 0.13	0.49 ± 0.07	11.2 ± 2.00
AVB + DVA + PVC (*N* = 10)	1.90 ± 0.28	1.03 ± 0.14	0.40 ± 0.21	12.2 ± 1.53
AVA + AVB + AVE + PVC (*N* = 10)	0.60 ± 0.21	0.39 ± 0.14	1.00 ± 0.12	8.66 ± 1.54

*Shown are population average times and standard errors of the mean (SEM) for worms in forward and backward motion, and in stopped phase. The last column gives population averages of reversal frequencies with their SEM*.

Worms with multiple ablations reverse roughly as frequently as worms with single ablations (Table 2). Generally, the frequency of direction reversals does not correlate well with the average time worms spent in forward motion (Table 2). For example, worms with killed ASH reverse approximately as often as worms with removed AVD, despite the fact that ASH worms spent three times more time in forward motion. Similarly, worms with almost equal *T*_*f*_ (about 0.9 s), i.e., AVB + PVC and AVA + AVB + PVC, differ in reversals by a factor of 2.5.

An interesting result is that ablating the modulatory neuron DVA causes a sharp decline in the forward motion timing in comparison to WT, and a two-fold increase in reversals (Table 2). Also, the combined ablations of DVA with PVC and AVB show a similar property. This clearly suggests that this neuron has a significant influence on the interneuron circuit output, which could be more than just its sensory modulation.

### Theoretical results

In our circuit model there are seven neurons (five pre-motor interneurons, DVA, and ASH), each of them can be either excitatory or inhibitory. Thus, there are 2^7^ = 128 possible copies of the circuit associated with synaptic polarities, i.e., the sign of ϵ_*i*_ (for *i* = 1, …, 7). Two examples of the polarity copies are: (1) ϵ_ASH_ = −1, ϵ_AVB_ = −1, ϵ_PVC_ = −1, ϵ_DVA_ = −1, ϵ_AVA_ = −1, ϵ_AVD_ = −1, ϵ_AVE_ = −1 and (2) ϵ_ASH_ = 1, ϵ_AVB_ = 1, ϵ_PVC_ = 1, ϵ_DVA_ = 1, ϵ_AVA_ = 1, ϵ_AVD_ = 1, ϵ_AVE_ = 1, which correspond to all inhibitory and all excitatory neurons, respectively. Additionally, each of the six pre-motor neurons (excluding ASH) receives an upstream sensory input coming mostly form the head, except for PVC for which it comes predominantly from the tail. The sensory input is represented by a binary variable *z*_*i*_ that can have two values: 0 for a weak input, and 1 for a strong input (see Equation 7 in Materials and Methods). Consequently, every polarity copy can be found in additional 2^6^ = 64 activity or input configurations. This implies that, in total, our circuit model has 2^7^ · 2^6^ = 8192 distinct polarity-input configurations.

For each possible configuration (i.e., synaptic polarity and an upstream input) of the circuit we performed 17 “computer ablations” analogous to the experimental ablations shown in Table 2, by setting ϵ_*i*_ = 0 if the neuron with an index *i* was removed. This procedure removes all the connections (synaptic and electric) coming out of this ablated neuron. For each ablation we computed the fraction of time corresponding to forward motion, i.e., *T*_*f*_/(*T*_*f*_ + *T*_*b*_), using Equation (8). Thus, we generated 18 theoretical fractional times associated with every circuit configuration (17 types of ablation plus WT) and computed their Euclidean distance (ED) to the experimental values in Table 2 (see Materials and Methods for a more detailed description). The configuration with the smallest ED value corresponds to the optimal solution that predicts synaptic polarities and the pattern of the upstream input. Our strategy was to vary the level of this input (σ, κ), and for each level to find optimal values of synaptic and gap junction conductances (*q*_*s*_, *q*_*e*_), together with the system noise amplitude (η), which give the best fit of the theoretical *T*_*f*_/(*T*_*f*_ + *T*_*b*_) to the experimental data.

#### Winning configurations

**Figure 2:**
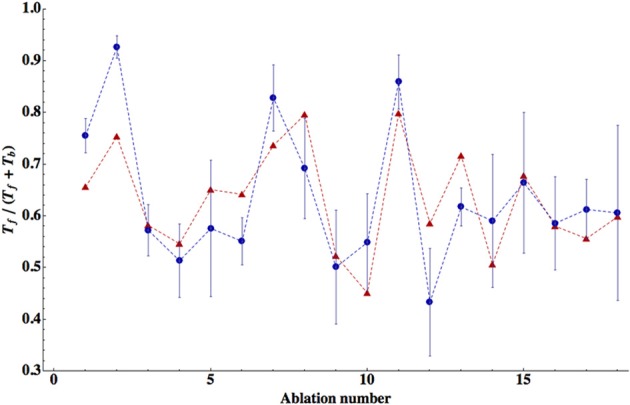
**Comparison of the theory with the data for relative times spent in forward locomotion across different ablations.** The theoretical values are for the winning polarity combination # 1, corresponding to all inhibitory neurons. Correlation between theoretical points (red triangles) and experimental (blue circles) is relatively high (*R* = 0.743) and statistically significant (*p* = 0.0004). The error bars for the experimental points were computed from SEM values of *T*_*f*_ and *T*_*b*_ given in Table 2. The optimal values of the free parameters are given in Table 3.

We find that the best match to the experimental data is achieved in the case when all seven neurons in the circuit are inhibitory (Tables 3–5). The winning synaptic polarity configuration is associated with the combination number 1, which gives the best (lowest) ED = 0.363, and the largest correlation with the data points, which is 0.743 (Table 3). The distribution of winning values of the upstream input *z*_*i*_ is non-homogeneous, and non-zero only for AVB and PVC neurons, implying that much sensory excitation comes to the neurons controlling directly forward motion (Table 3). In Figure 2 we display a comparison of theoretical and experimental values of *T*_*f*_/(*T*_*f*_ + *T*_*b*_) across different ablations for this winning combination of synaptic polarities. In general, there is a good fit of the theoretical points to the data (correlation about 0.74).

**Table 3:** **The winning combinations of interneuron polarities (ϵ_*i*_) for the upstream input: σ = 8.0 mV and κ = 0.6**.

**Neuron**	**Rank**								**Inhibitory likelihood**
	**1**	**2**	**3**	**4**	**5**	**6**	**7**	**8**	
ASH	−1	−1	−1	−1	−1	−1	−1	−1	1
AVA	−1	−1	−1	−1	−1	−1	1	1	3/4
AVB	−1	1	−1	1	1	−1	−1	−1	5/8
AVD	−1	−1	1	1	−1	−1	−1	−1	3/4
AVE	−1	−1	−1	−1	−1	−1	−1	−1	1
DVA	−1	−1	1	1	1	1	1	−1	3/8
PVC	−1	−1	−1	−1	−1	−1	−1	−1	1
Combination #	1	17	11	27	19	3	35	33	
ED	0.3625	0.3651	0.374	0.377	0.380	0.383	0.396	0.409	
Corr	0.7433	0.7417	0.722	0.717	0.740	0.746	0.690	0.731	

*The optimal values of the parameters are: η = 1.05 mV, q_*s*_ = 0.1 nS, and q_*e*_ = 0.1 nS. All the combinations receive the same “winning” input: z_AVB_ = 1, z_PVC_ = 1, and z_*i*_ = 0 for other interneurons*.

*The last column provides estimates of probabilities that a given neuron is inhibitory. In Tables 3–6, ED was computed using Equations (8) and (9)*.

**Table 4:** **The winning combinations of interneuron polarities (ϵ_*i*_) for the upstream input: σ = 6.0 mV and κ = 0.6**.

**Neuron**	**Rank**								**Inhibitory likelihood**
	**1**	**2**	**3**	**4**	**5**	**6**	**7**	**8**	
ASH	−1	−1	−1	−1	−1	−1	−1	−1	1
AVA	−1	−1	−1	−1	−1	−1	−1	−1	1
AVB	−1	1	−1	1	−1	1	1	1	3/8
AVD	−1	−1	1	1	−1	−1	1	1	1/2
AVE	−1	−1	−1	−1	−1	−1	−1	−1	1
DVA	−1	−1	1	1	1	1	−1	1	1/2
PVC	−1	−1	−1	−1	−1	−1	1	−1	7/8
Combination #	1	17	11	27	3	19	26	10	
ED	0.368	0.377	0.394	0.404	0.422	0.424	0.431	0.433	
Corr	0.734	0.722	0.687	0.665	0.692	0.669	0.627	0.637	

*The optimal values of the parameters are: η = 0.85 mV, q_s_ = 0.1 nS, and q_e_ = 0.1 nS. All the combinations receive the same “winning” input: z_AVB_ = 1, z_PVC_ = 1, and z_i_ = 0 for other interneurons. The last column provides estimates of probabilities that a given neuron is inhibitory*.

**Table 5:** **The winning combinations of interneuron polarities (ϵ_*i*_) for the upstream input: σ = 4.0 mV and κ = 0.6**.

**Neuron**	**Rank**								**Inhibitory likelihood**
	**1**	**2**	**3**	**4**	**5**	**6**	**7**	**8**	
ASH	−1	−1	−1	−1	−1	−1	−1	−1	1
AVA	−1	−1	−1	−1	−1	−1	−1	−1	1
AVB	−1	1	−1	−1	−1	1	1	1	1/2
AVD	−1	−1	1	1	1	1	1	1	1/4
AVE	−1	−1	−1	−1	−1	−1	−1	−1	1
DVA	−1	−1	1	−1	−1	1	−1	−1	3/4
PVC	−1	−1	−1	−1	1	−1	1	−1	3/4
Combination #	1	17	11	9	10	27	26	25	
ED	0.414	0.431	0.453	0.461	0.465	0.471	0.476	0.487	
Corr	0.644	0.606	0.560	0.613	0.530	0.512	0.485	0.578	

*The optimal values of the parameters are: η = 0.7 mV, q_s_ = 0.1 nS, and q_e_ = 0.1 nS. All the combinations receive the same “winning” input: z_AVB_ = 1 and z_i_ = 0 for other interneurons. The last column provides estimates of probabilities that a given neuron is inhibitory*.

The second place among all synaptic polarities is taken by the combination # 17, for which all neurons, except AVB, are inhibitory. This combination has ED value very close to that obtained by the winning combination # 1 (Table 3). Moreover, these two combinations appear the most often among the winners, also for other choices of parameters describing the sensory input (Tables 3–6). The third place is taken by a combination with the number 11, in which only two neurons are excitatory: AVD and DVA. This combination also appears quite often among the leading polarities, but its ED is in some separation from the two winning synaptic combinations 1 and 17.

**Table 6:** **The winning combinations of interneuron polarities (ϵ_*i*_) for the upstream input: σ = 12.0 mV and κ = 0.6**.

**Neuron**	**Rank**								**Inhibitory likelihood**
	**1**	**2**	**3**	**4**	**5**	**6**	**7**	**8**	
ASH	−1	−1	−1	−1	−1	−1	−1	−1	1
AVA	−1	−1	1	1	−1	−1	−1	−1	3/4
AVB	1	−1	1	−1	1	−1	−1	1	1/2
AVD	1	1	1	1	−1	−1	−1	−1	1/2
AVE	−1	−1	−1	−1	1	1	−1	−1	3/4
DVA	−1	−1	−1	−1	−1	−1	−1	−1	1
PVC	1	1	1	1	1	1	−1	−1	1/4
Combination #	26	10	58	42	22	6	1	17	
ED	0.383	0.386	0.390	0.392	0.393	0.394	0.397	0.398	
Corr	0.715	0.708	0.694	0.688	0.691	0.686	0.687	0.685	

*The optimal values of the parameters are: η = 0.85 mV, q_s_ = 0.1 nS, and q_e_ = 0.3 nS. All the combinations receive the same “winning” input: z_AVB_ = 1, z_PVC_ = 1, and z_i_ = 0 for other interneurons. The last column provides estimates of probabilities that a given neuron is inhibitory*.

#### Optimal solution depends both on synaptic and input configurations

**Figure 3:**
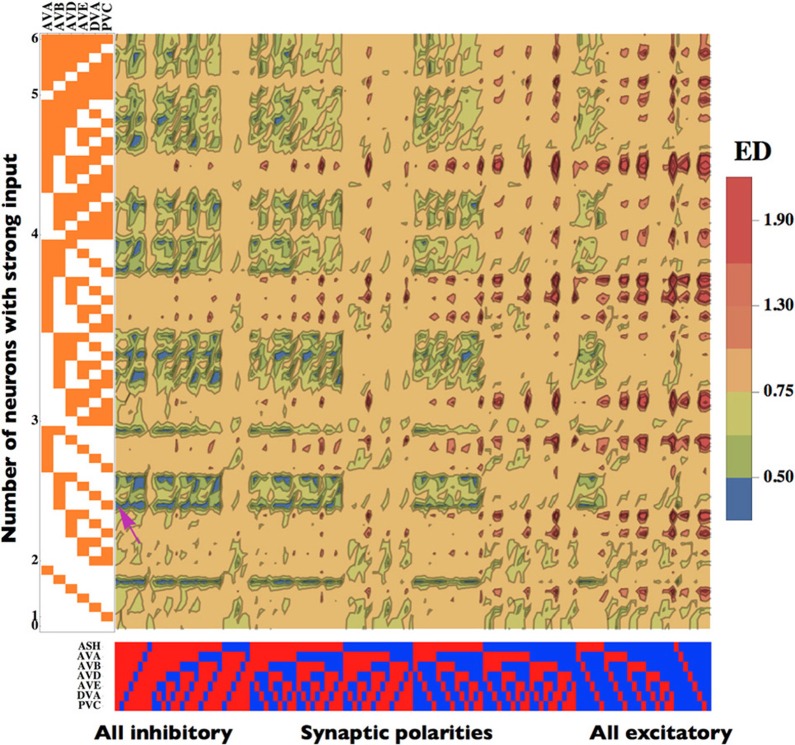
**Dependence of the Euclidean Distance (ED) on the patterns of synaptic polarities and input strength.** Neurons receiving a strong input are marked in orange. Inhibitory neurons are represented in red, while excitatory in blue. The smallest (optimal) value of ED is pinpointed by a pink arrow. Note that configurations with small ED are generally associated with mostly inhibitory connections and a moderate input strength (left part of the map), whereas large ED values occur for mostly excitatory configurations (right part of the map). The optimal parameters are the same as in Figure 2.

The degree of the match between theory and experiment, i.e., ED, depends not only on the pattern of synaptic polarities but also on the pattern of incoming sensory input (*z*_*i*_) to the network (Figure 3). We noticed that we obtained better fits if we allowed the interneurons to receive a heterogeneous input from upstream neurons. A given synaptic polarity configuration can produce a slightly different locomotory output (slightly variable ED) depending on how many, and which, interneurons receive a strong input (Figure 3). However, despite this subtlety the overall emerging picture is such that the smallest ED values are associated with configurations dominated by inhibitory connections, while the largests ED correspond to networks dominated by excitatory synapses, regardless of the input pattern. Among the configurations with the lowest ED, the most optimal are those with a moderate number (typically 2 or 3) of interneurons receiving strong input. The highest value of ED is about 2.0 and it occurs for synaptic configurations with prevalent excitatory connections, with only a minor influence of the input pattern (Figure 3). These results suggest that the ED is much more affected by the pattern of synaptic polarities than by the input pattern.

#### Likelihood estimates of interneuron synaptic polarities

To quantify the likelihood of a given synaptic polarity among leading combinations we associate with each neuron a probability that it is inhibitory, for each input value σ and ASH activity level (see last columns in Tables 3–6). This probability is defined here as a fraction of times the ϵ = −1 appears in the row for each neuron. One can notice that some of the locomotory interneurons, such as AVE, AVA, and PVC, are inhibitory with probabilities that are close to 1, regardless of the input magnitude. The polymodal neuron ASH is also in this category. For the rest of the pre-motor interneurons these probabilities are not that high, but nevertheless are at least ≥ 0.5. We also computed an average probability that a given neuron is inhibitory across different input levels coming to the locomotory circuit (average values for all Tables 3–6). These average probabilities are: 1 for ASH, 0.875 for AVA, 0.5 for AVB, 0.5 for AVD, 0.938 for AVE, 0.656 for DVA, and 0.719 for PVC. Thus, from the whole group of seven neurons investigated here and implicated in locomotion control, only AVB and AVD have about equal chances to be excitatory and inhibitory.

**Figure 4:**
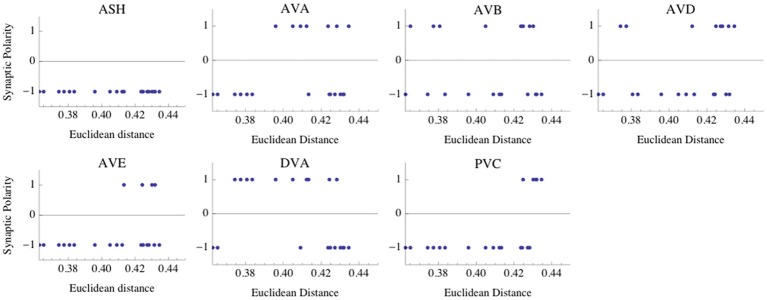
**Distribution of synaptic polarities for each interneuron.** The first 20 polarity combinations with the smallest Euclidean distance (ED) are shown, and they are associated with the optimal parameters given in Table 3. Note that the interneurons ASH, AVA, AVE, and PVC are inhibitory with a high probability. There are some non-zero likelihoods that AVB, AVD, and DVA neurons are excitatory (especially AVB and AVD), although the smallest ED values are associated with negative polarities. The optimal parameters are the same as in Figure 2.

One might wonder if these probability estimates hold if we include more winning combinations, not just eight as in Tables 3–6. Including 20 leading combinations for the winning input values σ = 8 mV and κ = 0.6 (corresponding to Table 3), gives qualitatively a similar picture. Again, the neurons AVB and AVD have the highest likelihood of being excitatory, although inhibitory polarities of these interneurons have the lowest ED values (Figure 4). Taken together, the above results strongly suggest that the majority of interneuron connections are inhibitory.

#### Dependence of ED on model free parameters

The ED between theoretical and experimental fractions of time spent in forward locomotion depends in a non-monotonic manner on model free parameters: *q*_*s*_, *q*_*e*_, σ, κ, and η. The first four parameters are neurophysiological in nature and determine the overall balance between currents flowing in the interneuron network. The last parameter, η characterizes a shape of the transfer function between neural activities and behavioral locomotory output, or in other words it characterizes a network noise level.

**Figure 5:**
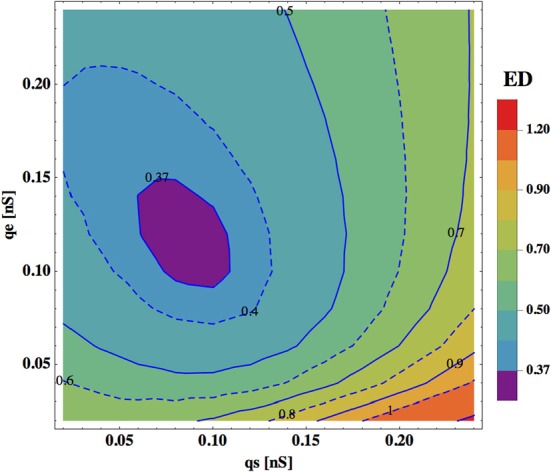
**Dependence of ED on synaptic and electric conductances.** ED is optimal (minimal) for some range of values of *q*_*s*_ and *q*_*e*_. Note that the changes in synaptic conductance (horizontal direction) are more critical for ED than the changes in *q*_*e*_ (vertical direction). The optimal values of other parameters are: σ = 8.0 mV, κ = 0.6, and η = 1.05 mV.

In Figure 5 we show a dependence of ED on synaptic and electric conductances (*q*_*s*_ and *q*_*e*_) in the system. In general, ED has a minimum for a narrow range of these conductances, indicating that an optimal solution exists for this range. A qualitatively similar picture emerges also for the rest of the free parameters. Typically, the parameters controlling the strength of the upstream input, i.e., σ and κ, should be in some intermediate range to reach a minimal value of ED. For instance, the winning polarity combinations for σ = 4 and σ = 12 mV have larger ED than that for σ = 6 or 8 mV (Tables 3–6). The same is true for the value of κ, characterizing ASH activity (see Materials and Methods), with the optimal κ = 0.6 for each σ level.

**Figure 6:**
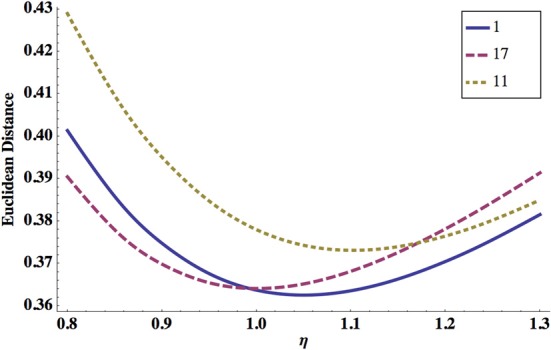
**Dependence of ED on the system noise amplitude η.** ED has a minimum for some optimal η, and this is essentially independent of the synaptic polarity configuration. Shown are synaptic configurations number 1 (solid line), 17 (dashed line), and 11 (dotted line). The optimal values of other parameters are: σ = 8.0 mV, κ = 0.6, and *q*_*s*_ = *q*_*e*_ = 0.1 nS.

In Figure 6 we show ED as a function of noise level η. Again, either too strong or too weak η increases ED, and there is an optimal value of this parameter for which ED has a minimum.

## Discussion

### The main findings

Using a combination of experimental (laser ablations) and computational (circuit model and optimization) tools we were able to decipher the likely synaptic signs of the interneurons composing the small network commanding *C. elegans* locomotion (Chalfie et al., 1985; White et al., 1986). It turns out that probably most of these neurons, i.e., synapses they sent, are inhibitory. In particular, the average probabilities that a particular interneuron is inhibitory are: 0.875 for AVA, 0.5 for AVB, 0.5 for AVD, 0.938 for AVE, 0.719 for PVC, and 0.656 for DVA. These numbers suggest that although some of the connections coming out of these neurons might be excitatory, the majority of the connections are clearly inhibitory.

Because of a suppressing nature, the command interneuron circuit must receive a sufficient amount of excitation from upstream (in large part sensory) neurons to be functional, i.e., to appropriately activate downstream motor neurons. Our computational results indicate that the behavioral data are best explained if the command circuit receives a mixed, heterogeneous input [denoted by *X*_*i*_ in our model; Equations (6) and (7)]. The best fit to the data is obtained if the largest excitation comes to forward interneurons AVB and PVC (Table 3; Figure 3). In this sense, the existence of sensory stimulation is an important factor for directional motion generation, which is in general agreement with the experimental findings (Zheng et al., 1999; Piggott et al., 2011).

### Role of interneuron gap junctions in locomotion

How are downstream motor neurons activated given an inhibitory nature of synapses in the pre-motor interneuron circuit? This probably occurs due to strong gap junction couplings between two major interneurons, AVB and AVA, and the downstream motor neurons of type A and B. Our results suggest that the worm moves forward because AVB receives a stronger upstream input than the “backward” interneurons. AVB then excites downstream B motor neurons via strong gap junctions. The chemical synapses from AVB to B are weak and thus non-significant (Table 7). Therefore the sign of these synapses is irrelevant for forward motion, and AVB does not necessarily have to be excitatory.

**Table 7:** **Connectivity matrix for the command interneuron circuit**.

**Postsynaptic neuron**	**Presynaptic neurons**								
	**ASH**	**AVA**	**AVB**	**AVD**	**AVE**	**DVA**	**PVC**	**F**	**B**
AVA	1.75	–	6.75	15.75	10.5	2.0	5.0	–	0.25
	–	–	–	–	–	–	(2.5)	(3.5)	(25.5)
AVB	2.25	0.5	–	0.25	–	0.5	7.75	–	–
	–	–	–	–	–	(1.0)	–	(13.75)	(0.5)
AVD	3.0	1.0	0.75	–	0.25	–	3.25	–	0.25
	–	–	–	–	–	–	–	–	–
AVE	0.75	1.0	0.75	–	–	7.0	1.25	–	–
	–	–	–	–	–	–	–	–	–
DVA	–	–	–	–	–	–	2.0	0.5	–
	–	–	(1.0)	–	–	–	(0.5)	(0.5)	–
PVC	–	7.0	–	0.25	0.25	2.0	–	0.25	1.25
	–	(2.5)	–	–	–	(0.5)	–	(0.75)	(0.75)
F	–	2.5	0.25	0.25	0.25	6.5	–	–	–
	–	(3.5)	(13.75)	–	–	(0.5)	(0.75)	–	–
B	–	41.75	1.5	7.0	8.25	1.0	1.0	–	–
	–	(25.5)	(0.5)	–	–	–	(0.75)	–	–

*Shown are average anatomical number of synapses (N_s, ij_) and gap junctions (N_e, ij_) (in the brackets below synaptic contacts) between postsynaptic neuron i and presynaptic neuron j. Symbols F and B denote forward and backward motor neurons, respectively*.

The issue with backward motion is more subtle. Recent calcium imaging studies show that AVA is active during backward movement (Chronis et al., 2007; Faumont et al., 2011). How can one explain this? The likely answer lies again in the strong gap junction connections between AVA and A motor neurons, and between AVB and B motor neurons (Table 7). Specifically, during backward motion AVA, due to its large sensory input, synaptically inhibits other interneurons including AVB, but at the same time excite downstream A motor neurons via strong electric coupling. Thus AVB sends less excitation to B motor neurons via its strong gap junctions than does AVA to A neurons. Consequently, the activity of A prevails over the activity of B neurons (i.e., *E*_*b*_ > *E*_*f*_ in our model), and the worm moves backward, even with inhibitory synapses in the locomotor circuit.

### Relative importance of the patterns of synaptic polarity and sensory input on the results

Our results indicate that locomotory output of the interneuron circuit depends on its synaptic polarities as well as on sensory input pattern. This can be seen in Figure 3, where the ED characterizing the degree of the match between the theory and the data is displayed as a function of synaptic and input configurations. From these two main factors affecting ED, the synaptic polarity pattern seems to be a more important determinant. This is because the lowest values of ED are generally associated with circuit configurations in which synaptic inhibition dominates, whereas the highest ED's are related to mostly excitatory connections (Figure 3). On the other hand, the sensory input pattern does not exhibit such a simple general trend. More precisely, increasing the amount of strong input in the network does not lead to an explicit decrease (or increase) of ED, but rather to its fluctuations. Instead, ED assumes the smallest values for a moderate number of interneurons receiving strong input, which is usually 2 or 3 (Figure 3).

### Properties of ASH and AVB neurons strongly affect the results

From all neurons present in our locomotory circuit, two neurons ASH and AVB are particularly important for the network performance. This is strongly related to the question of specificity in synaptic polarity and input pattern. The map in Figure 3 shows that the synaptic polarity of ASH is strongly correlated with the level of ED. In most cases, synaptic configurations in which ASH is inhibitory decrease ED, and increase it if ASH is excitatory. The exceptions are configurations with small inputs, where the opposite takes place. Thus, for the most part the synaptic polarity of ASH should be inhibitory in terms of the network optimality.

AVB neuron, which provides the main signal to the downstream motor neurons controlling forward locomotion, is also critical for the optimal solutions. The strength of the sensory input this neuron receives strongly correlates with ED (Figure 3). Generally, strong input in AVB decreases ED, whereas weak input in AVB increases ED, and this effect is essentially independent on synaptic polarity configurations (Figure 3). This suggest that on average AVB should receive a strong excitatory input coming from upstream neurons.

If we combine the action of both ASH and AVB we obtain an interesting picture. ED values are the smallest for cases in which ASH is inhibitory and AVB receives a strong input (Figure 3). In an opposite case, when ASH is excitatory and AVB gets a weak input, ED values are the largests.

### Dependence of network performance on model free parameters

Performance (ED) of the interneuron circuit depends also on several neurophysiological parameters and on the level of noise in the system. Generally, these parameters require fine-tuning to obtain the best fit between the theory and the data. Synaptic conductance *q*_*s*_ and gap junction conductance *q*_*e*_ determine the relative strength of synaptic and electric connections. Optimal values of these parameters that give the best model performance are always in a physiological range, and yield very similar values ~0.1 nS. However, ED is more sensitive to changes in synaptic conductance *q*_*s*_ than to changes in *q*_*e*_ (ED has a broad minimum as a function of *q*_*e*_; Figure 5), which suggests that precise values of synaptic conductance are more important.

ED has always a minimum as a function of noise amplitude, regardless of the values of other parameters (Figure 6). This optimal value of noise is in the range 0.7–1.1 mV (Tables 3–6), which seems to agree with the magnitude for voltage fluctuations obtained from electrophysiology (Piggott et al., 2011). The optimality of the system performance for some finite level of noise resembles a phenomenon known as a stochastic resonance, which is a ubiquitous mechanism in many physical and biological systems (McDonnell and Abbott, 2009).

The circuit performance not only depends on the pattern of the sensory input (see above), but also on the strength of this input (Tables 3–6). There exists some optimal level of upstream excitation for which ED has a minimum. Either increase or decrease of this level has a negative influence on the system performance. This non-monotonic dependence can be explained in the following way. When the incoming sensory input is too strong its contribution to an interneuron voltage is much bigger than the contributions coming from synaptic and gap junction couplings. Thus manipulation of parameters associated with connectivity does not change the network output, and one cannot improve the system performance. On the other hand some minimal input is required to stimulate the network, so the signal flows to the motor neurons.

The level of excitation in ASH neuron, controlled by κ, can be also viewed as a measure of an additional input coming to the interneuron circuit. Therefore, a non-monotonic dependence of ED on κ should not be a surprise, which is what we observe in our computational analysis.

### Asymmetry in forward and backward interneuron activities determine the likely direction of motion

Our results indicate that a decision to move in a particular direction can be made on a small circuit level composed of the six pre-motor interneurons (including DVA). Specifically, the output from these interneurons is fed to the two types of body wall motor neurons (B and A) controlling forward and backward motions, whose relative asymmetric activities (*E*_*f*_ and *E*_*b*_ in our model) determine the likely direction of worm's movement. This simple decision making mechanism can explain 74% of the correlations between the experimental data and computational results (see Table 3). Moreover, this behavioral picture is consistent with the findings in a recent experimental study (Kawano et al., 2011), in which it is shown that the imbalance between activities of A and B motor neurons is a likely scenario for the selection of worm's motion direction. However, in contrast to these authors the imbalance between A and B neurons in our model is caused not so much by a strong AVA-A gap junction coupling, but by the asymmetric upstream excitatory input to command interneurons (in the winning combinations, AVB and PVC neurons receive much stronger upstream excitation than the rest of the circuit).

### Qualitative interpretation of ablation data

The interesting experimental result concerning single-neuron ablations is that removal of certain interneurons causes an increase in forward motion timing, while removal of others leads to its dramatic decrease. Specifically, only killings of ASH and PVC neurons increase significantly the time *T*_*f*_ spent in forward locomotion. In relation to ASH, this suggests an important role of the sensory input. There are two surprises here. First, PVC was thought as promoting forward locomotion (Chalfie et al., 1985). Second, given that the sensory ASH neuron makes only weak or intermediate synaptic connections with the command interneurons (with all “backward” interneurons and AVB; see Table 7), it should not have such a strong influence on the motion characteristics. The solution of these puzzles is that PVC and ASH are probably inhibitory, i.e., the strongest synapses connecting these neurons with other postsynaptic targets should be inhibitory. Additionally, ASH should be highly depolarized in order to significantly downregulate the locomotory (mostly backward) interneurons via its synapses. Recent calcium imaging indicates that ASH to AVA synapse is likely excitatory (Chronis et al., 2007). This experimental result does not necessarily contradict our result regarding the negative ASH polarity. Indeed, our results concern only an average total polarity of a given neuron, and it is possible that some of its weak synapses can have a reverse polarity in relation to the strongest. In fact, the ASH to AVA synapse is relatively weak (third in strength out of four coming out of ASH; see Table 7), and it could be dominated by stronger inhibitory synapses to other neurons.

Removal of AVA interneuron causes a large reduction in *T*_*f*_, despite the fact that this neuron belongs to the “backward” locomotory circuit, and one might naively expect that it effectively prohibits forward motion. Moreover, single and multiple ablations associated with AVA cause an increase in stopped time (Table 2). This suggests that removal of AVA decreases a difference between activities of forward and backward motor neurons, i.e., *E*_*f*_ − *E*_*b*_ may become comparable with a threshold for movement initiation Δ (see Supplementary Information). This in turn may suggest that when AVA is absent, backward motor neurons are more active. Taken together, these results imply that the overall synaptic effect of AVA is most likely inhibitory.

The ablation results for the DVA neuron indicate that it plays a more significant role in the locomotory circuit than just its passive modulation. From Table 2 it is evident that killing DVA has one of the biggest impacts on *T*_*f*_. Based on this, we hypothesize that DVA might serve also as a command neuron in generation of forward locomotion, which is a novel function for this neuron.

The case with multiple ablations is more complicated. These type of ablations do not have an additive property, i.e., removal of more neurons does not necessarily lead to a progressive drop in the forward motion timings. For example, double AVB and PVC ablation has *T*_*f*_ = 0.91 s, but additional removal of AVD actually increases *T*_*f*_ to 1.33 s (Table 2). The latter may seem paradoxical, however, one has to remember that backward neurons do not act in isolation, but participate in the whole interneuron network activity, and thus indirectly also influence the forward motor neurons. Apart from that, interneurons interact among themselves both synaptically (non-linear in nature) and via gap junctions (bidirectional in nature). This may additionally mask a single interneuron contribution to the locomotory output of the circuit. As a result it is very difficult to predict in advance the effect of any particular ablation on worm's locomotory characteristics in the case of multiple ablations. For this, one needs to perform detailed computations on a network level, as was executed in this study.

### Collective, mutually inhibitory interactions between command interneurons underlie *C. elegans* direction of locomotion

These ablation results suggest that a picture in which a single neuron or a single connection control a specific behavior, advocated in several former studies (Chalfie et al., 1985; Gray et al., 2005), may be oversimplified. Instead, our findings support the idea that behavioral (locomotory) output depends to a large degree on a collective activity of neurons comprising the “functional circuit” (Zheng et al., 1999). That is, the same neuron can participate to some extent in opposite behaviors. Obviously, some neurons or connections in the functional circuits may be more dominant than others for a particular behavior, but the presence or absence of a particular neuron in the circuit is generally not critical for its operation. This partial redundancy in neural function is probably evolutionary driven to ensure a robust circuit performance.

Similarly, none of the interneuron ablations, either single or multiple, abolishes the worm's movement or body oscillations completely. This clearly indicates that none of the interneurons alone is a Central Pattern Generator, which again speaks in favor of collective rather than individual interneuron dynamics as a determinant of locomotion.

Our main result that the pre-motor interneuron circuit has mainly inhibitory synapses is qualitatively similar to two earlier (Wicks et al., 1996; Zheng et al., 1999) and one recent (Qi et al., 2012) study. Wicks et al. (1996) investigated computationally a tap withdrawal circuit in *C. elegans* and predicted that most interneurons composing it were inhibitory (Wicks et al., 1996). That study concluded that PVC and AVD interneurons were probably excitatory. Our results suggest that AVD is equally likely to be inhibitory as excitatory, whereas PVC with high probability should be inhibitory. The possible sources of the discrepancy can be that Wicks et al. (1996) used an older incomplete connectivity data for the pre-motor interneurons (White et al., 1986), did not include AVE neuron, and used a little different set of neurons in their analysis. In another study, Zheng et al. (1999) hypothesized that the locomotory interneuron circuit should act as an inhibitory switch in order to explain qualitatively data on motion direction transitions. A recent experimental work (Qi et al., 2012) also suggests that the pre-motor interneurons should use inhibition as a main synaptic signaling.

An interesting question is which neurotransmitters mediate inhibitory interactions between interneurons. The most likely neurotransmitter between interneurons is glutamate. In mammalian brains, it is known to be exclusively an excitatory signal, since AMPA and NMDA postsynaptic receptors conduct mostly Na^+^ and K^+^ with an effective reversal potential around 0 mV. However, in *C. elegans* the situation is more complicated because locomotor interneurons contain apart from these receptors, also GluCl postsynaptic receptors (Brockie and Maricq, 2006). These channels are gated by Cl^−^ (with large negative reversal potentials), and therefore mediate inhibition to postsynaptic cells (Brockie and Maricq, 2006). Specifically, the currents associated with GluCl receptor have been observed in the AVA interneuron (Mellem et al., 2002), and they may also exist in other interneurons.

Given these two types of postsynaptic receptors, it is possible that a single interneuron can have both excitatory and inhibitory synapses on distinct postsynaptic targets. In this case, the synaptic polarities associated with each neuron in our study have an average character. More precisely, the determined probabilities that a given neuron is inhibitory are the fractions of inhibitory connections that the neuron makes with other postsynaptic neurons. Thus, for example, the inhibitory probability 0.5 found for AVB indicates that this neuron sends out about 50% inhibitory and 50% excitatory synapses.

### Our computational model and its extension

The theoretical approach in this paper blends a traditional neural network modeling with a probabilistic method for relating network activity to behavioral data. In particular, we envision the nematode locomotion as a three state system, one state for forward, second for backward motion, and third state for no motion. In this system there are transitions between the states that are caused by intrinsic relative activities of A and B motor neurons (*E*_*b*_ and *E*_*f*_), as well as by the system noise (η in Equation 8). Note that many ablations in Table 2 have the ratio *T*_*f*_/*T*_*b*_ close to unity, which in terms of our model implies that (*E*_*f*_ − *E*_*b*_)/η ≪ 1, i.e., for these ablations a stochastic influence of the environment is bigger than the relative activities of A and B motor neurons. This is interesting and shows that sensory noise gains in importance as we remove more neurons from our circuit. This may also suggest that some of the interneurons act as filters for the environmental noise.

Our combined approach allows us to achieve a concrete goal, which is the prediction of synaptic polarities for the well defined locomotory interneuron circuit and the determination of the likely sensory input pattern. This prediction does not depend on a precise form of the transfer function between neural activation and locomotory output (compare Equations 15 and 16 in the Supplementary Information). In this sense our results are robust.

The knowledge of the probable synaptic polarities of the command interneurons may have a positive impact on future modeling studies of *C. elegans* locomotion. We hope, that this will enable more realistic simulations of the neuronal dynamics that can extend the scope of testable predictions of the current locomotory models (Karbowski et al., 2006, 2008; Bryden and Cohen, 2008). Our method of determining synaptic polarities, which combines structural perturbations with the computational modeling, is sufficiently general that can be also applied to other small functional neural systems in which synaptic polarities are unknown. However, it is important to keep in mind that our model, as every model describing biology, is subject to several assumptions (see the list in the Materials and Methods), and clearly has some limitations. The model does not include several subtle neurophysiological features. For example, a possibility that an individual neuron might release multiple neurotransmitters of similar importance, or that neuromodulators might provide an extra synaptic input throughout the network. These features can be addressed in future more detailed investigations.

## Materials and methods

The ethics statement does not apply to this study.

### Collection of experimental data

#### Strain maintenance

For our automated locomotion analysis, we cultured *C. elegans* at 20°C on NGM plates seeded with the OP50 using standard methods (Brenner, 1974).

#### Automated worm tracking and data extraction

Worms tested by automated tracking were continuously cultured on E. coli OP50. For assaying various parameters of locomotion, 10 cm non-seeded NGM plates were used. These NGM plates used for recordings were equilibrated to 20°C for 18–20 h. After ablations of individual neurons, the worms were placed on plates with *E .coli* as a food source to recover. Ablated worms and mock controls were tested within 72 h of the L4 molt. They were then transferred to assay plates containing no food. After 5 min of acclimatization on these plates, the worms were video taped for 5 min. Data presented in this paper represent the locomotory behavior of worms when they were exhibiting “area restricted search behavior”. Data extraction and processing was done using image processing and analysis software as previously described (Cronin et al., 2005). From each video recording of 5 min, we used the middle 4 min, and used the software to derive values for times in forward and backward locomotion, as well as reversal frequencies. In our software, we used a velocity threshold for motion detection. Specifically, if the nematode velocity was below 0.05 mm/s, we classified this as stopped time or “no motion”. Every change of velocity direction that was above this value was classified as a reversal. The average time spent in the stopped phase is *T*_*s*_. The time *T*_*f*_ the worm spent in forward motion is defined as an average duration of time counted from a moment of moving forward to stopping. Similarly, the time *T*_*b*_ spent in backward motion is an average of times from the initiation of backward movement to stopping. Generally, because of many reversals, the sum of the times *T*_*f*_, *T*_*b*_, and *T*_*s*_ is much smaller than the recording time of 4 min. The numerical values of *T*_*f*_, *T*_*b*_, *T*_*s*_ provided in this paper are population averages. All incubations and recordings were done in a constant temperature room at 20°C.

#### Laser ablations

For all species tested, we used the L1 larva stage for our ablations.

### Description of the command interneuron circuit model

#### List of the assumptions used to construct the model

We make the following major assumptions in the theoretical model:

(1) In the interneuron circuit left and right members of each interneuron are grouped as one interneuron.

(2) Synaptic and gap junction strengths between any two neurons are proportional to the anatomical number of synapses and gap junctions between them.

(3) Pre-motor neurons do not generate sodium-type action potentials but their activities are graded, as *C. elegans* genome lacks molecules coding for voltage-activated sodium channels (Bargmann, 1998). This assumption is also consistent with electrophysiological observations in *C. elegans* and related nematodes (Davis and Stretton, 1989; Goodman et al., 1998).

(4) Each neuron releases a single neurotransmitter, or equivalently, there exists a dominant neurotransmitter type for each neuron. Thus, with each neuron in the command circuit we can associate a single dominant synaptic polarity.

(5) Worm's movement direction is determined by a relative imbalance in the activities of excitatory motor neurons of type A and B, which is in agreement with recent experimental observations (Kawano et al., 2011).

(6) Behavioral output of the worm can be formally described in terms of a three-state model. The three states correspond to forward motion, backward motion, and stopped period. Each state has its probability of occurrence, which for forward and backward states is given by an exponential function of a difference between activities of type A and B motor neurons (see below).

#### Derivation of the model equations

Equations describing interneuron circuit responsible for forward–backward motion transitions are given below. This is a non-linear model based on synaptic connectivity data from www.wormatlas.org [updated version of White et al. (1986) wiring diagram from Chen et al. (2006)].

We start with a standard membrane equation describing the graded dynamics of neuron with an index *i* (Ermentrout and Terman, 2010):

(1) $$\begin{array}{l} CS\frac{dV_{i}}{dt}=-g_{L}S\left(V_{i}-V_{r}\right)-\sum_{j}g_{s,\;ij}H_{0}\left(V_{j}\right)\left(V_{i}-V_{s,\;j}\right)\\ \;-\sum_{j}g_{e,\;ij}\left(V_{i}-V_{j}\right) \end{array}$$

where *V*_*i*_ is the voltage of neurons *i*, *C* is the membrane capacitance per surface area, *S* is the total surface area of neuron *i*, *V*_*r*_ is the resting voltage, *g*_*L*_ is the total membrane ionic conductance per surface area that is composed mainly of a constant leak current (typical K^+^ channel conductance is much smaller for voltages close to *V*_*r*_), *g*_*e*, *ij*_ is the gap junction conductance between neurons *i* and *j*. The symbol *g*_*s*, *ij*_ denotes synaptic conductance coming from *j* presynaptic neuron with synaptic reversal potential *V*_*s*, *j*_. The function **H_0_**(*V*_*j*_) is a non-linear sigmoidal function characterizing synaptic transmission, and is given by

(2) $$H_{0}\left(V_{j}\right)=\frac{1}{1+\exp\left[-\gamma \left(V_{j}-\theta_{0}\right)\right]},$$

where θ_0_ is the voltage threshold for synaptic activation, and γ is a measure of steepness of the activation slope. Generally, the synaptic input strongly depends on γ. We set its value at 0.15 mV^−1^ following an earlier analysis (Wicks et al., 1996) (Table 1). This particular value yields realistic synaptic currents that cause changes in voltage membrane by at most several mV, which agrees with known neurophysiology in other animals (Koch, 1998).

Our goal is to write Equation (1) in a more convenient form for the investigation of synaptic polarities. We assume that the resting potential *V*_*r*_ (when no synaptic or gap junction input is present) for *C. elegans* interneurons is −40 mV, which agrees with earlier suggestions (Wicks et al., 1996), and it is close to a recent measurement (≈ −50 mV) in AIB neuron (Piggott et al., 2011). We want to re-define the voltage in Equation (1) as a deviation from its resting value, i.e., we introduce Δ*V*_*i*_ ≡ *V*_*i*_ − *V*_*r*_. Let's denote by *V*_ex_ the reversal potential for excitatory, and by *V*_in_ the reversal potential for inhibitory synapses. The value of *V*_ex_ is around 0 mV (the current in excitatory synapses is mediated by Na^+^, K^+^, and partly by Ca^++^). The value of *V*_in_ was reported between −70 mV (Purves et al., 2008) and −90 mV (Koch, 1998) (the current in inhibitory synapses is mediated by Cl^−^). As an average for *V*_in_ we take −80 mV. Consequently, we obtain for excitatory synapses *V*_*i*_ − *V*_ex_ = Δ*V*_*i*_ + *V*_*r*_ − *V*_ex_ = Δ*V*_*i*_ − 40, and for inhibitory *V*_*i*_ − *V*_in_ = Δ*V*_*i*_ + *V*_*r*_ − *V*_in_ = Δ*V*_*i*_ + 40. The resulting average numerical factors in both expressions have identical absolute values. Thus we can use an approximation: *V*_*i*_ − *V*_*s*,*j*_ ≈ Δ*V*_*i*_ − ϵ_*j*_A, where *A* = 40 mV, and ϵ_*j*_ characterizes the synaptic polarity of the presynaptic neuron *j*. The value of ϵ_*j*_ is either 1 for excitatory synapses or −1 for inhibitory. Taking the above into account and dividing both sides of Equation (1) by *g*_*L*_S, we can rewrite this equation as

(3) $$\frac{C}{g_{L}}\frac{d(\Delta V_{i})}{dt}=-\left[1+\sum_{j}\frac{g_{s,\;ij}H_{0}\left(V_{j}\right)}{g_{L}S}\right]\Delta V_{i}+\sum_{j}\epsilon_{j}A\frac{g_{s,\;ij}}{g_{L}S}H_{0}\left(V_{j}\right)-\sum_{j}\frac{g_{e,\;ij}}{g_{L}S}\left(\Delta V_{i}-\Delta V_{j}\right).$$

We can determine the strengths of synaptic and gap junction connections between any *i* and *j* interneurons by their anatomical numbers *N*_*s*, *ij*_, *N*_*e*, *ij*_, and maximal elementary conductances *q*_*s*_, *q*_*e*_, i.e., *g*_*s*, *ij*_ = *N*_*s*, *ij*_*q*_*s*_, and *g*_*e*, *ij*_ = *N*_*e*, *ij*_*q*_*e*_. The data for *N*_*s*, *ij*_ and *N*_*e*, *ij*_ are available from the data set in Chen et al. (2006) (see Table 7). A typical range of conductances for chemical and electrical synapses is known from neurophysiology of other animals (Koch, 1998). The leak conductance *g*_*L*_ is taken as *g*^−1^_*L*_ = 150 kΩ· cm^2^ (Wicks et al., 1996), which comes form the neurophysiological measurements in a related larger nematode *Ascaris* (Davis and Stretton, 1989). The surface area *S* of all interneurons is very similar and around 15 · 10^−6^ cm^2^ (White et al., 1986; Wicks et al., 1996), so we obtain *g*_*L*_S = 0.1 nS, of which the inverse (i.e., 10^10^ Ω) is comparable to an experimental measurement of the total input resistance ~ 0.5 · 10^10^ Ω (Goodman et al., 1998). Consequently, we can estimate the ratios present in Equation (3) as: *Ag*_*s*, *ij*_/(*g*_L_*S*) = 400 *N*_*s*, *ij*_*q*_*s*_, and *g*_*e*, *ij*_/(*g*_L_*S*) = 10 *N*_*e*, *ij*_*q*_*e*_, where *q*_*s*_, *q*_*e*_ are expressed in nS. We checked that the term ∑_*j*_ *g*_*s*, *ij*_**H**(*V*_*j*_)/(*g*_L_*S*), which is associated with Δ*V*_*i*_ is generally much smaller than 1, since **H**(*V*_*j*_) ≪ 1 for voltages not far away from *V*_*r*_. Consequently, this term is neglected, which simplifies significantly the resulting equations for interneurons (see below and the Supplementary Information). Thus, we can write Equation (3) in an approximate form as:

(4) $$\tau \frac{d(\Delta V_{i})}{dt}\approx -\Delta V_{i}+\sum_{j}\epsilon_{j}w_{ij}H\left(\Delta V_{j}\right)-\sum_{j}g_{ij}\left(\Delta V_{i}-\Delta V_{j}\right),$$

where τ = *C*/*g*_*L*_ is the membrane time constant, *w*_*ij*_ is the synaptic coupling *w*_*ij*_ = 400*q*_*s*_ *N*_*s*, *ij*_, and *g*_*ij*_ is the gap junction coupling *g*_*ij*_ = 10*q*_*e*_ *N*_*e*, *ij*_. The function **H**(Δ*V*_*j*_) in Equation (4) differs from the original function **H_0_**(*V*_*j*_) only by a substitution θ_0_ → θ, where θ = θ_0_ − *V*_*r*_, i.e.,

(5) $$H(\Delta V_{j})=\frac{1}{1+\exp\left[-\gamma \left(\Delta V_{j}-\theta \right)\right]}.$$

It is important to keep in mind that synaptic polarities in Equation (4) are determined simply by the signs of ϵ_*j*_ coefficients.

#### Activity equations for interneurons

Equations describing activities of the interneurons in the locomotory circuit are presented in the Supplementary Information. They are similar in form to Equation (4) with an additional inclusion of the heterogeneous excitatory sensory input *X*_*i*_, i.e.,

(6) $$\tau \frac{d(\Delta V_{i})}{dt}=-\Delta V_{i}+\sum_{j}\epsilon_{j}w_{ij}H\left(\Delta V_{j}\right)+\;\sum_{j}\epsilon_{i}^{2}\epsilon_{j}^{2}g_{ij}\left(\Delta V_{j}-\Delta V_{i}\right)+X_{i},$$

where the subscripts *i*, *j* are labels for our circuit neurons. The parameter ϵ_*i*_ denotes synaptic polarity of the neuron *i*, and it assumes value 1 (if the neuron is excitatory), value −1 (if inhibitory), or 0 (if the neuron is absent because of the ablation). Ablations in the circuit remove also electric connections if a neuron on either side of this coupling is killed. To include this effect we rescale the gap junction couplings by ϵ^2^_*i*_ϵ^2^_*j*_ factors. The square in the epsilon assures that we do not get unphysical negative values for gap junction conductance. We assume that the excitatory sensory input *X*_*i*_ coming from the upstream neurons to the interneuron *i* is constant in time and represented by

(7) $$X_{i}=x_{0}+\sigma z_{i},$$

where *x*_0_ = 2.0 mV, σ is a variable parameter characterizing the strength of a strong input that is constant in time, and *z*_*i*_ is the binary variable either 0 (weak input) or 1 (strong input). Thus neurons can receive only two types of the input: either weak (*x*_0_) or strong (*x*_0_ + σ). The “input” parameter *z*_*i*_, similar to ϵ_*i*_, is unknown. We want to find their optimal values for each interneuron.

The above pre-motor interneurons make synaptic and gap junction connections with downstream excitatory motor neurons. Two separate groups of these motor neurons generating forward and backward motion, called B and A, respectively, directly connect locomotory muscles. The activities of excitatory motor neurons (*E*_*f*_ and *E*_*b*_ for forward and backward motion, respectively) have a similar form to that of the interneurons and are given in the Supplementary Information. Generally, activity equations for interneurons and motor neurons are of similar kind to those used before in Karbowski et al. (2008) for analyzing forward locomotion.

We solve Equations (6, 7) using a second order Runge–Kutta method. We are interested only in the steady-state activities of the interneurons and motor neurons. One can think about these activities as temporal averages over sufficiently long periods of time driven by a constant bias input. This simplifying step significantly enhances the feasibility of the analysis. The steady-state values of motor neuron activities, *E*_*f*_ and *E*_*b*_, are inserted in Equation (8); see below. All the results presented were obtained by choosing initial conditions corresponding to resting voltages, i.e., Δ*V*_*i*_ = 0 for each neuron. We also tried random initial conditions in which neurons start with voltages uniformly distributed in the range −10 to 10 mV. In both cases the steady-state values are the same, which means that steady-state activities are independent of initial conditions, at least in that range.

#### Theoretical ablations

Ablations or removals of neurons in the model are performed by setting ϵ_*neuron*_ = 0. For example, if we remove neuron AVB, then we put ϵ_*AVB*_ = 0 in all equations for neural activities.

#### Values of the connectivity matrix

The strength *w*_*ij*_ of synaptic input to neuron *i* coming from neuron *j* is given according to Equation (4) by the expression *w*_*ij*_ = 400*q*_*s*_ *N*_*s*, *ij*_, where *N*_*s*, *ij*_ is the number of synaptic contacts of neuron *i* with presynaptic neuron *j*. The number *N*_*s*, *ij*_ is determined as an arithmetic mean for the right- and left-hand side interneurons. As an example, the right AVB neuron receives an input from both right and left PVC neurons, of which we take an arithmetic mean. Similarly, the left AVB neuron receives an input from both right and left PVC, of which we again take an arithmetic mean. Next, we take an arithmetic mean of these two arithmetic means, and obtain a single value representing average number of synaptic contacts between presynaptic PVC and postsynaptic AVB (*N*_*s*,AVB,PVC_). The strength of gap junction *g*_*ij*_ between neurons *i* and *j* is given by an analogous formula *g*_*ij*_ = 10*q*_*e*_ *N*_*e*, *ij*_, where *N*_*e*, *ij*_ is the number of gap junction contacts between *i* and *j* (arithmetic mean of right and left interneurons). Empirical data for *N*_*s*, *ij*_ and *N*_*e*, *ij*_ were taken from the database in Chen et al. (2006) and are presented in Table 7. Parameters *q*_*s*_ and *q*_*e*_ were taken in the range: *q*_*s*_ = 0.03 − 0.6 nS and *q*_*e*_ = 0.03 − 0.5 nS (Koch, 1998).

#### ASH neuron

From all upstream neurons we selected explicitly ASH neuron because of its polymodal sensory role. Specifically, it has been implicated in avoidance responses (Kaplan and Horvitz, 1993), which are associated with the modulation of locomotion. We do not write an explicit equation for ASH dynamics, because it receives a massive input form many other head neurons, of which we have no knowledge. Instead, we make computations for four different levels of ASH activity that we set by hand. We choose *ASH* = κθ, where κ = 0.2, 0.4, 0.6, or 0.75. The value of the normalized threshold θ is set to 45 mV.

#### Relationship to the behavioral data

From the experimental part we have average times the worms spent in forward and backward locomotion, which we denote as *T*_*f*_ and *T*_*b*_, respectively. These average times should be somehow related to the average activities of the two type of motor neurons, *E*_*f*_ and *E*_*b*_. The precise relationship between them is unknown due to the lack of direct physiological data. However, one can expect that domination of *E*_*f*_ over *E*_*b*_ should favor forward motion and its duration, and vice versa.

We can make some progress by using an analogy with statistical physics (Gardiner, 2004), and treating worm's locomotory behavior as a three state system influenced by both deterministic and stochastic factors. These three states correspond to forward movement, backward movement, and stopped time (no motion). There could be transitions between the states driven by sensory input from the environment (either deterministic or stochastic). However, we do not model such transitions. We are interested only in long-term “average” or steady states activities of the system. Our model is motivated in large part by experimental results of Kawano et al. (2011). In that study it was shown that *C. elegans* motion direction is determined by a relative activity of A and B motor neurons. In particular, it was suggested (Kawano et al., 2011) that when forward motor neurons are much more active than backward (i.e., *E*_*f*_ ≫ *E*_*b*_), then there should be a high probability of finding the worm in the forward motion. Conversely, if the activity of backward motor neurons dominates over the activities of their forward counterparts (i.e., *E*_*b*_ ≫ *E*_*f*_), then there is a high chance that the worm moves backward. Thus, it appears that the sign of *E*_*f*_ − *E*_*b*_ plays a key role in the choice of worm's motion direction. Moreover, one can expect that when the activities of both types of motor neurons are comparable (equal or almost equal), then *C. elegans* likely does not move.

We relate the behavioral observables, i.e., the fraction of time spent in forward motion, with the motor neuron activities using a transfer function known as a sigmoidal logistic function. Specifically, we propose

(8) $$\frac{T_{f}}{\left(T_{f}+T_{b}\right)}=\frac{1}{1+\exp\left[\left(E_{b}-E_{f}\right)/\eta \right]},$$

where η is the noise in the system. This parameter also determines the shape of the transfer function. This type of function is a standard tool often used in many computational studies in neuroscience, and this is the primary transfer function used in this study. Note that for cases in which activities of A motor neurons dominate, i.e., (*E*_*b*_ − *E*_*f*_)/η ≫ 1, the time spent in forward motion is relatively short, i.e., *T*_*f*_/*T*_*b*_ ≪ 1. The derivation of Equation (8) is presented in the Supplementary Information.

#### The goal

We want to determine which combination of neuron polarities: ϵ_ASH_, ϵ_AVB_, ϵ_PVC_, ϵ_DVA_, ϵ_AVA_, ϵ_AVD_, ϵ_AVE_, together with their corresponding upstream inputs *z*_*i*_, yields the best fit to the experimental values of *T*_*f*_/(*T*_*f*_ + *T*_*b*_).

#### Comparison of the theory with the data

In our model there are 8192 distinct combinations of synaptic polarities ϵ_*i*_ and the upstream inputs *z*_*i*_, i.e., different configurations in which the circuit can be found. For each circuit configuration, we perform 17 interneuron ablations in our computer model, and compute theoretical values of *T*_*f*_/(*T*_*f*_ + *T*_*b*_) for each ablation. Next, we compute an ED of these values to the experimental values given in Table 2. The ED serves as a system performance (the lower ED the better), and it is computed according to the expression:

(9) $$\text{ED}={\left[\sum_{a\;=\;1}^{18}{\left(R_{\text{th}}-R_{\text{ex}}\right)}_{a}^{2}\right]}^{1/2},$$

where *R* = *T*_*f*_/(*T*_*f*_ + *T*_*b*_) ≡ (*T*_*f*_/*T*_*b*_)[1 + *T*_*f*_/*T*_*b*_]^−1^, and the subscripts th and ex refer to theoretical and experimental values of *R*. The subscript *a* refers to the ablation number, in the same order as in Table 2. In particular, *a* = 1 corresponds to the mock ablation, i.e., WT. All the results presented in Tables 3–6 were generated by using Equation (8) for the parameter *R*_*th*_.

### Conflict of interest statement

The authors declare that the research was conducted in the absence of any commercial or financial relationships that could be construed as a potential conflict of interest.

## References

[B1] Bargmann C. I. (1998). Neurobiology of the *Caenorhabditis elegans* genome. Science 282, 2028–2033 10.1126/science.282.5396.2028 9851919

[B2] Brenner S. (1974). The genetics of Caenorhabditis elegans. Genetics 77, 71–94 4366476 10.1093/genetics/77.1.71 PMC1213120

[B3] Brockie P. J. Maricq A. V. (2006). Ionotropic glutamate receptors: genetics, behavior, and electrophysiology, in Wormbook, eds The C. elegans Research Community Available online at: http://www.wormbook.org 10.1895/wormbook.1.61.1 PMC4781458 18050468

[B4] Bryden J. A. Cohen N. (2008). Neural control of *Caenorhabditis elegans* forward locomotion: the role of sensory feedback. Biol. Cybern. 98, 339–351 10.1007/s00422-008-0212-6 18350313

[B5] Chalfie M. Sulston J. E. White J. G. Southgate E. Thomson J. N. Brenner S. (1985). The neural circuit for touch sensitivity in *Caenorhabditis elegans*. J. Neurosci. 5, 956–964 3981252 10.1523/JNEUROSCI.05-04-00956.1985 PMC6565008

[B6] Chen B. L. Hall D. H. Chklovskii D. B. (2006). Wiring optimization can relate neuronal structure and function. Proc. Natl. Acad. Sci. U.S.A. 103, 4723–4728 10.1073/pnas.0506806103 16537428 PMC1550972

[B7] Cherniak C. (1994). Component placement optimization in the brain. J. Neurosci. 14, 2418–2427 8158278 10.1523/JNEUROSCI.14-04-02418.1994 PMC6577144

[B8] Chklovskii D. B. (2004). Synaptic connectivity and neuronal morphology: two sides of the same coin. Neuron 43, 609–617 10.1016/j.neuron.2004.08.012 15339643

[B9] Chronis N. Zimmer M. Bargmann C. I. (2007). Microfluidics for *in vivo* imaging of neuronal and behavioral activity in *Caenorhabditis elegans*. Nat. Methods 4, 727–731 10.1038/nmeth1075 17704783

[B10] Cronin C. J. Mendel J. E. Mukhtar S. Kim Y. M. Stirbl R. C. Bruck J. (2005). An automated system for measuring parameters of nematode sinusoidal movement. BMC Genet. 6:5 10.1186/1471-2156-6-5 15698479 PMC549551

[B11] Davis R. E. Stretton A. O. W. (1989). Passive membrane properties of motorneurons and their role in long-distance signaling in the nematode *Ascaris*. J. Neurosci. 9, 403–414 2918369 10.1523/JNEUROSCI.09-02-00403.1989 PMC6569814

[B12] de Bono M. Maricq A. V. (2005). Neuronal substrates of complex behaviors in *C. elegans*. Annu. Rev. Neurosci. 28, 451–501 10.1146/annurev.neuro.27.070203.144259 16022603

[B13] DeFelipe J. Alonso-Nanclares L. Avellano J. (2002). Microstructure of the neocortex: Comparative aspects. J. Neurocytol. 31, 299–316 10.1023/A:1024130211265 12815249

[B14] Ermentrout G. B. Terman D. H. (2010). Mathematical Foundations of Neuroscience. New York, NY: Springer 10.1007/978-0-387-87708-2

[B15] Faumont S. Rondeau G. Thiele T. R. Lawton K. J. McCormick K. E. Sottile M. (2011). An image-free opto-mechanical system for creating virtual environments and imaging neuronal activity in freely moving *Caenorhabditis elegans*. PLoS ONE 6:e24666 10.1371/journal.pone.0024666 21969859 PMC3182168

[B16] Gardiner C. W. (2004). Handbook of Stochastic Methods. 3rd Edn Berlin: Springer

[B17] Goodman M. B. Hall D. H. Avery L. Lockery S. R. (1998). Active currents regulate sensitivity and dynamic range in *C. elegans* neurons. Neuron 20, 763–772 10.1016/S0896-6273(00)81014-4 9581767 PMC4444786

[B18] Gray J. M. Hill J. J. Bargmann C. I. (2005). A circuit for navigation in *Caenorhabditis elegans*. Proc. Natl. Acad. Sci. U.S.A. 102, 3184–3191 10.1073/pnas.0409009101 15689400 PMC546636

[B19] Ha H. Hendricks M. Shen Y. Gabel C. V. Fang-Yen C. Qin Y. (2010). Functional organization of a neural network for aversive olfactory learning in *Caenorhabditis elegans*. Neuron 68, 1173–1186 10.1016/j.neuron.2010.11.025 21172617 PMC3038580

[B20] Haider B. Duque A. Hasenstaub A. R. McCormick D. A. (2006). Neocortical network activity *in vivo* is generated through a dynamic balance of excitation and inhibition. J. Neurosci. 26, 4535–4545 10.1523/JNEUROSCI.5297-05.2006 16641233 PMC6674060

[B21] Haspel G. O'Donovan M. J. Hart A. C. (2010). Motorneurons dedicated to either forward or backward locomotion in the nematode *C. elegans*. J. Neurosci. 30, 11151–11156 10.1523/JNEUROSCI.2244-10.2010 20720122 PMC2945236

[B22] Hobert O. (2003). Behavioral plasticity in *C. elegans*: paradigms, circuits, genes. J. Neurobiol. 54, 203–223 10.1002/neu.10168 12486705

[B23] Jospin M. Qi Y. Stawicki T. Boulin T. Schuske K. Horvitz H. (2009). A neuronal acetylcholine receptor regulates the balance of muscle excitation and inhibition in *Caenorhabditis elegans*. PLoS Biol. 7:e1000265 10.1371/journal.pbio.1000265 20027209 PMC2787625

[B24] Kaiser M. Hilgetag C. C. (2006). Nonoptimal component placement, but short processing paths, due to long-distance projections in neural systems. PLoS Comput. Biol. 2:e95 10.1371/journal.pcbi.0020095 16848638 PMC1513269

[B25] Kaplan J. M. Horvitz H. R. (1993). A dual mechanosensory and chemosensory neuron in *Caenorhabditis elegans*. Proc. Natl. Acad. Sci. U.S.A. 90, 2227–2231 10.1073/pnas.90.6.2227 8460126 PMC46059

[B26] Karbowski J. (2001). Optimal wiring principle and plateaus in the degree of separation for cortical neurons. Phys. Rev. Lett. 86, 3674–3677 10.1103/PhysRevLett.86.3674 11328051

[B27] Karbowski J. (2003). How does connectivity between cortical areas depend on brain size? Implications for efficient computations. J. Comput. Neurosci. 15, 347–356 10.1023/A:1027467911225 14618069

[B28] Karbowski J. Cronin C. J. Seah A. Mendel J. E. Cleary D. Sternberg P. W. (2006). Conservation rules, their breakdown, and optimality in *Caenorhabditis* sinusoidal locomotion. J. Theor. Biol. 242, 652–669 10.1016/j.jtbi.2006.04.012 16759670

[B29] Karbowski J. Schindelman G. Cronin C. J. Seah A. Sternberg P. W. (2008). Systems level circuit model of *C. elegans* undulatory locomotion: mathematical modeling and molecular genetics. J. Comput. Neurosci. 24, 253–276 10.1007/s10827-007-0054-6 17768672

[B30] Kawano T. Po M. D. Gao S. Leung G. Ryu W. S. Zhen M. (2011). An imbalancing act: gap junctions reduce the backward motor circuit activity to bias *C. elegans* forward locomotion. Neuron 72, 572–586 10.1016/j.neuron.2011.09.005 22099460

[B31] Koch C. (1998). Biophysics of Computation. Oxford: Oxford University Press

[B32] Li W. Feng Z. Sternberg P. W. Xu S. X. Z. (2006). A *C. elegans* stretch receptor neuron revealed by a mechanosensitive TRP channel homologue. Nature 440, 684–687 10.1038/nature04538 16572173 PMC2865900

[B33] McDonnell M. D. Abbott D. (2009). What is stochastic resonance? Definitions, misconceptions, debates, and its relevance to biology. PLoS Comput. Biol. 5:e1000348 10.1371/journal.pcbi.1000348 19562010 PMC2660436

[B34] Mellem J. E. Brockie P. J. Zheng Y. Madsen D. M. Maricq A. V. (2002). Decoding of polymodal sensory stimuli by postsynaptic glutamate receptors in *C. elegans*. Neuron 36, 933–944 10.1016/S0896-6273(02)01088-7 12467596

[B35] Piggott B. J. Liu J. Feng Z. Wescott S. A. Xu X. Z. S. (2011). The neural circuits and synaptic mechanisms underlying motor initiation in *C. elegans*. Cell 147, 922–933 10.1016/j.cell.2011.08.053 22078887 PMC3233480

[B36] Purves D. Augustine G. J. Fitzpatrick D. Hall W. C. LaMantia A. S. McNamara J. O. (2008). Neuroscience, 4th Edn. Sunderland, MA: Sinauer Associates

[B37] Qi Y. B. Garren E. J. Shu X. Tsien R. Y. Jin Y. (2012). Photo-inducible cell ablation in Caenorhabditis elegans using the genetically encoded singlet oxygen generating protein miniSOG. Proc. Natl. Acad. Sci. U.S.A. 109, 7499–7504 10.1073/pnas.1204096109 22532663 PMC3358873

[B38] Sengupta P. Samuel A. D. T. (2009). *Caenorhabditis elegans*: a model system for systems neuroscience. Curr. Opin. Neurobiol. 19, 637–643 10.1016/j.conb.2009.09.009 19896359 PMC2904967

[B39] van Vreeswijk C. Sompolinsky H. (1996). Chaos in neuronal networks with balanced excitatory and inhibitory activity. Science 274, 1724–1726 10.1126/science.274.5293.1724 8939866

[B40] Varshney L. R. Chen B. L. Paniagua E. Hall D. H. Chklovskii D. B. (2011). Structural properties of the *Caenorhabditis elegans* neuronal network. PLoS Comput. Biol. 7:e1001066 10.1371/journal.pcbi.1001066 21304930 PMC3033362

[B41] Vogels T. P. Sprekeler H. Zenke F. Clopath C. Gerstner W. (2011). Inhibitory plasticity balances excitation and inhibition in sensory pathways and memory networks. Science 334, 1569–1573 10.1126/science.1211095 22075724

[B42] White J. G. Southgate E. Thomson J. N. Brenner S. (1986). The structure of the nervous system of the nematode *Caenorhabditis elegans*. Philos. Trans. R. Soc. Lond. B 314, 1–340 10.1098/rstb.1986.0056 22462104

[B43] Wicks S. R. Roehrig C. J. Rankin C. H. (1996). A dynamic network simulation of the nematode tap withdrawal circuit: predictions concerning synaptic function using behavioral criteria. J. Neurosci. 16, 4017–4031 8656295 10.1523/JNEUROSCI.16-12-04017.1996 PMC6578605

[B44] Zheng Y. Brockie P. J. Mellem J. E. Madsen D. M. Maricq A. V. (1999). Neuronal control of locomotion in *C. elegans* is modified by a dominant mutation in the GLR-1 ionotropic glutamate receptor. Neuron 24, 347–361 10.1016/S0896-6273(00)80849-1 10571229

