# Supplementary material for: Synaptic polarity of the interneuron circuit controlling C. elegans locomotion
Source: Front Comput Neurosci. 2013 Oct 2;7:128. doi: 10.3389/fncom.2013.00128 (PMC3788333; doi:10.3389/fncom.2013.00128)
Supplement: Supplementary file 1 [file Presentation1.PDF]

Supplementary information for:

Synaptic polarity of the interneuron circuit controlling *C. elegans* locomotion

Franciszek Rakowski[a], Jagan Srinivasan[b], Paul W. Sternberg[b], and Jan Karbowski[c,d]

[a] *Interdisciplinary Center for Mathematical and Computational Modeling, University of Warsaw, 02-106 Warsaw, Poland*

[b] *California Institute of Technology, Division of Biology, Pasadena, CA 91125, USA*

[c] *Institute of Applied Mathematics and Mechanics, University of Warsaw, 02-097 Warsaw, Poland*

[d] *Institute of Biocybernetics and Biomedical Engineering, Polish Academy of Sciences, 02-109 Warsaw, Poland*

Activity equations for interneurons.

The dynamics of the interneurons in the locomotory circuit are based on the derived Eq. (4) in the main text. They are represented by a following set of differential equations:

$$\begin{aligned}\tau\frac{dAVB}{dt} &= -AVB + \epsilon_{ASH}w_{AVB,ASH}\mathbf{H}(ASH) + \epsilon_{PVC}w_{AVB,PVC}\mathbf{H}(PVC)\\ &+\epsilon_{AVA}w_{AVB,AVA}\mathbf{H}(AVA) + \epsilon_{AVD}w_{AVB,AVD}\mathbf{H}(AVD)\\ &+\epsilon_{DVA}w_{AVB,DVA}\mathbf{H}(DVA) + \epsilon^2_{AVB}\epsilon^2_{DVA}g_{AVB,DVA}(DVA-AVB)\\ &+\epsilon^2_{AVB}g_{AVB,F}(E_f - AVB) + \epsilon^2_{AVB}g_{AVB,B}(E_b - AVB) + X_{AVB}, \end{aligned} \quad (1)$$

$$\begin{aligned}\tau\frac{dPVC}{dt} &= -PVC + \epsilon_{AVA}w_{PVC,AVA}\mathbf{H}(AVA) + \epsilon_{DVA}w_{PVC,DVA}\mathbf{H}(DVA)\\ &+\epsilon_{AVD}w_{PVC,AVD}\mathbf{H}(AVD) + \epsilon_{AVE}w_{PVC,AVE}\mathbf{H}(AVE)\\ &+w_{PVC,F}\mathbf{H}(E_f) + w_{PVC,B}\mathbf{H}(E_b)\\ &+\epsilon^2_{PVC}\epsilon^2_{AVA}g_{PVC,AVA}(AVA-PVC) + \epsilon^2_{PVC}\epsilon^2_{DVA}g_{PVC,DVA}(DVA-PVC)\\ &+\epsilon^2_{PVC}g_{PVC,F}(E_f - PVC) + \epsilon^2_{PVC}g_{PVC,B}(E_b - PVC) + X_{PVC}, \end{aligned} \quad (2)$$

$$
\begin{aligned}
\tau\frac{dDVA}{dt} &= -DVA + \epsilon_{PVC}w_{DVA,PVC}\mathbf{H}(PVC) + w_{DVA,F}\mathbf{H}(E_f) \\
&+\epsilon^2_{DVA}\epsilon^2_{AVB}g_{DVA,AVB}(AVB - DVA) + \epsilon^2_{DVA}\epsilon^2_{PVC}g_{DVA,PVC}(PVC - DVA) \\
&+\epsilon^2_{DVA}g_{DVA,F}(E_f - DVA) + X_{DVA}, \qquad (3)
\end{aligned}
$$

$$
\begin{aligned}
\tau\frac{dAVA}{dt} &= -AVA + \epsilon_{ASH}w_{AVA,ASH}\mathbf{H}(ASH) + \epsilon_{AVB}w_{AVA,AVB}\mathbf{H}(AVB) \\
&+\epsilon_{PVC}w_{AVA,PVC}\mathbf{H}(PVC) + \epsilon_{AVD}w_{AVA,AVD}\mathbf{H}(AVD) \\
&+\epsilon_{AVE}w_{AVA,AVE}\mathbf{H}(AVE) + \epsilon_{DVA}w_{AVA,DVA}\mathbf{H}(DVA) \\
&+w_{AVA,B}\mathbf{H}(E_b) + \epsilon^2_{AVA}\epsilon^2_{PVC}g_{AVA,PVC}(PVC - AVA) \\
&+\epsilon^2_{AVA}g_{AVA,B}(E_b - AVA) + \epsilon^2_{AVA}g_{AVA,F}(E_f - AVA) + X_{AVA}, \qquad (4)
\end{aligned}
$$

$$
\begin{aligned}
\tau\frac{dAVD}{dt} &= -AVD + \epsilon_{AVB}w_{AVD,AVB}\mathbf{H}(AVB) + \epsilon_{PVC}w_{AVD,PVC}\mathbf{H}(PVC) \\
&+\epsilon_{ASH}w_{AVD,ASH}\mathbf{H}(ASH) + \epsilon_{AVA}w_{AVD,AVA}\mathbf{H}(AVA) \\
&+\epsilon_{AVE}w_{AVD,AVE}\mathbf{H}(AVE) + w_{AVD,B}\mathbf{H}(E_b) + X_{AVD}, \qquad (5)
\end{aligned}
$$

$$\tau \frac{dAVE}{dt} = -AVE + \epsilon_{AVB} w_{AVE,AVB} \mathbf{H}(AVB) + \epsilon_{PVC} w_{AVE,PVC} \mathbf{H}(PVC) + \epsilon_{DVA} w_{AVE,DVA} \mathbf{H}(DVA) + \epsilon_{ASH} w_{AVE,ASH} \mathbf{H}(ASH) + \epsilon_{AVA} w_{AVE,AVA} \mathbf{H}(AVA) + X_{AVE}, \tag{6}$$

where AVB, PVC, DVA, AVA, AVD, AVE are the relative activities of the corresponding interneurons with respect to their resting values. The parameters w_{ij} are synaptic strengths, and g_{ij} are gap-junction (electrical) couplings, where the subscripts i and j denote the above interneurons. The symbol ϵ_i denotes synaptic polarity of the neuron i, and it assumes value 1 (if the neuron is excitatory), value -1 (if inhibitory), or 0 (if the neuron is absent because of the ablation). Note that gap junction couplings contain the ϵ_i^2 factors, which are either 1 (if the neuron i is present), or 0 (if it is removed from the network). The parameter X_i describes a constant in time input coming from the upstream neurons to the interneuron i. For all interneurons except PVC this input comes from the head neurons. It is represented by $X_i = x_0 + \sigma z_i$, where $x_0 = 2.0$ mV, σ is a variable parameter, and z_i is either 0 (weak input) or 1 (strong input). The parameter z_i, similar to ϵ_i, is unknown. We want to find both of them for each interneuron.

The above pre-motor interneurons make synaptic and gap junction connections with downstream excitatory motor neurons. Two separate groups of these motor neurons generating forward and backward motion, called B and A respectively, directly connect locomotory muscles. The activities of excitatory motor neurons are given by:

$$
\begin{aligned}
\tau\frac{dE_f}{dt} &= -E_f + \epsilon_{PVC}w_{F,PVC}\mathbf{H}(PVC) + \epsilon_{DVA}w_{F,DVA}\mathbf{H}(DVA) \\
&+\epsilon_{AVA}w_{F,AVA}\mathbf{H}(AVA) + \epsilon_{AVB}w_{F,AVB}\mathbf{H}(AVB) \\
&+\epsilon_{AVD}w_{F,AVD}\mathbf{H}(AVD) + \epsilon_{AVE}w_{F,AVE}\mathbf{H}(AVE) \\
&+\epsilon^2_{AVB}g_{F,AVB}(AVB - E_f) + \epsilon^2_{AVA}g_{F,AVA}(AVA - E_f) \\
&+\epsilon^2_{PVC}g_{F,PVC}(PVC - E_f) + \epsilon^2_{DVA}g_{F,DVA}(DVA - E_f),
\end{aligned}
\tag{7}
$$

and

$$
\begin{aligned}
\tau\frac{dE_b}{dt} &= -E_b + \epsilon_{AVA}w_{B,AVA}\mathbf{H}(AVA) + \epsilon_{AVD}w_{B,AVD}\mathbf{H}(AVD) \\
&+\epsilon_{AVE}w_{B,AVE}\mathbf{H}(AVE) + \epsilon_{AVB}w_{B,AVB}\mathbf{H}(AVB) \\
&+\epsilon_{PVC}w_{B,PVC}\mathbf{H}(PVC) + \epsilon_{DVA}w_{B,DVA}\mathbf{H}(DVA) \\
&+\epsilon^2_{AVA}g_{B,AVA}(AVA - E_b) + \epsilon^2_{AVB}g_{B,AVB}(AVB - E_b) + \epsilon^2_{PVC}g_{B,PVC}(PVC - E_b),
\end{aligned}
\tag{8}
$$

where E_f and E_b are the total relative activities of forward (type B) and backward (type A) motor neurons, respectively, measured from their resting voltages.

Three state model of *C. elegans* locomotion.

We assume that *C. elegans* locomotion can be described approximately as a three state model. These three states correspond to forward movement, backward movement,

and stopped time (no motion). The average times the worm spends in each state are denoted as T_f, T_b, and T_s, respectively. With each state we associate probabilities of occurance, as is explained below. The probability of forward motion P_f can be written as

$$P_f = Z \exp[(E_f - E_b - \Delta)/\eta_0], \tag{9}$$

where Δ is some activity threshold for locomotion initiation, the parameter η_0 characterizes the level of noise in the system, and Z is the normalization factor. By symmetry considerations, we can write the probability for backward motion P_b as

$$P_b = Z \exp[(E_b - E_f - \Delta)/\eta_0]. \tag{10}$$

The choice of the exponential function in P_f and P_b is motivated by two major arguments. First, with the exponentials both P_f and P_b are always increasing and positive functions of the arguments $E_f - E_b$ and $E_b - E_f$ for the whole range of their variabilities (from $-\infty$ to $+\infty$), which is generally not the case for other simple choices, in particular polynomials. For example, the choice $P_f \sim (E_f - E_b - \Delta)^n$, with n an even integer, is not satisfactory because the probability P_f is a non-monotonic function of its argument (it has a minimum for $E_f = E_b + \Delta$). Similarly, if the exponent n is an odd integer, then P_f becomes negative for $E_f < E_b + \Delta$, which is clearly wrong. A second argument in favor of the exponential function in the probabilities is the fact that many

phenomena occurring in nature have a similar type of dependence.

The probability that the nematode does not move, i.e. it is in the stopped state, is

$$P_s = Z \cdot S_s \left(\frac{E_f - E_b}{\Delta} \right), \tag{11}$$

where $S_s(x)$ is some unknown function that should have the following properties. For $|x| \ll 1$, i.e. when $|E_f - E_b|$ is much smaller than the motion activation threshold Δ, the function $S_s(x) \gg 1$. For $|x| \gg 1$, i.e. when either E_f or E_b dominates, the worm should not be motionless, which corresponds to $S_s(x) \to 0$. Additionally, due to symmetry one should have $S_s(-x) = S_s(x)$. The form of the $S_s(x)$ function is however irrelevant for the kind of computations made in this paper (see below).

From the normalization condition for probabilities, $P_f + P_b + P_s = 1$, we can determine Z, which allows us to find explicit forms for the state probabilities. The latter read:

$$P_f = \frac{e^{(E_f - E_b - \Delta)/\eta_0}}{e^{(E_f - E_b - \Delta)/\eta_0} + e^{(E_b - E_f - \Delta)/\eta_0} + S_s} \tag{12}$$

$$P_b = \frac{e^{(E_b - E_f - \Delta)/\eta_0}}{e^{(E_f - E_b - \Delta)/\eta_0} + e^{(E_b - E_f - \Delta)/\eta_0} + S_s} \tag{13}$$

$$P_s = \frac{S_s}{e^{(E_f - E_b - \Delta)/\eta_0} + e^{(E_b - E_f - \Delta)/\eta_0} + S_s} \tag{14}$$

In a case when the activity of forward motor neurons dominates over the rest ($E_f \gg E_b + \Delta$), we have $P_f \to 1$, $P_b \to 0$, and $P_s \to 0$. For a balanced activation of forward and backward motor neurons, i.e. when $E_f \approx E_b$, we have $S_s \gg 1$, which leads to $P_f \approx P_b \ll 1$, and $P_s \to 1$.

On the other hand the probabilities P_f, P_b, and P_s are related to the times (T_f, T_b, T_s) the worm spends in the corresponding states. The average probability that *C. elegans* is in the forward state is $P_f = T_f/(T_f + T_b + T_s)$. Similarly, $P_b = T_b/(T_f + T_b + T_s)$, and $P_s = T_s/(T_f + T_b + T_s)$. Thus, the ratio T_f/T_b is equal to P_f/P_b. Combining the above equations, we obtain

$$T_f/T_b = \exp\left[(E_f - E_b)/\eta\right], \tag{15}$$

where $\eta = \eta_0/2$. Note that the ratio of times associated with forward and backward locomotion neither depends on the activation threshold Δ nor on the form of the $S_s(x)$ function. The ratio of times depends only on the difference in the activation of complementary motor neurons and the level of noise in the system. It is interesting to stress that the quantity of empirical interest, i.e. $T_f/(T_f+T_b)$ is equal to $[1 + \exp((E_b - E_f)/\eta)]^{-1}$. The latter expression is known as a sigmoidal logistic function, and serves as a transfer

function from neural activities to behavioral output.

In order to examine the robustness of our results, we also investigated another choice for T_f/T_b as a function of $E_f - E_b$, different from that given by Eq. (8) in the main text. Specifically, we tried the following function:

$$T_f/T_b = \ln(1 + e^{(E_f - E_b)/\eta})/\ln 2. \quad (16)$$

Note that for $E_b \gg E_f$, Eqs. 15 and 16 both yield T_f/T_b that are proportional to each other. For $E_f \gg E_b$ they behave differently, i.e. the ratio T_f/T_b increases with $E_f - E_b$ much faster in Eq. (15) (exponential) than in Eq. (16) (linear). We found that the winning combinations had essentially the same ED values for both choices of T_f/T_b, which implies that Eqs. (15) and (16) yield equivalent results for the best configurations. This means that our results are not sensitive to the precise form of the transfer function between neural activities and locomotory output.
